# The association between childhood trauma and facial emotion recognition in women with premenstrual dysphoric disorder

**DOI:** 10.1192/j.eurpsy.2025.577

**Published:** 2025-08-26

**Authors:** A. Vardiampasis, C. Gramandani

**Affiliations:** 1 Mental Health Center, General Hospital of Rethymno, Crete, Rethymno, Greece

## Abstract

**Introduction:**

Facial emotion recognition is a fundamental component in social interaction. Facial emotion recognition is disturbed both in women with Premenstrual Dysphoric Disorder (PMDD), and in those with a history of childhood trauma. PMDD affects up to 5% of women of childbearing age, exert influence on women’s recognition of emotions, and on the emotion recognition processing. Women with PMDD are more likely to have a history of childhood trauma.

**Objectives:**

To explore whether there is a link between a history of trauma and the perception of emotions in women with PMDD. We hypothesize that women with PMDD and a history of childhood trauma will show larger deficits in emotion recognition compared to women with PMDD but without a history of childhood trauma.

**Methods:**

Data were derived from a sample of forty women diagnosed with PMDD (18-30 y.o., right handed, educational level >9y., regular cycle duration), who have visited Mental Health Centre of Rethymno (participants completed the Premenstrual Syndrome Questionnaire). The participants completed the Childhood Trauma Questionnaire (CTQ), which measures five types of maltreatment experiences. Three types are related to abuse (physical, sexual and emotional) and two to physical and emotional neglect. The Emotion Recognition Task (ERT) was also administered. ERT is a computer-generated paradigm for measuring the recognition of six basic facial emotional expressions: anger, disgust, fear, happiness, sadness, and surprise. During this test, video clips of increasing length are presented.

**Results:**

The majority of the participants (82.5%) reported a history of maltreatment during childhood. Women without trauma, when they completed the ERT did not show any significant emotion dysregulation. On the contrary, maltreated women, especially physically or sexually abused, had a distorted perception of emotions expressed on adult faces. Happiness is less detected, whereas fear and anger are recognized more rapidly and at a lower intensity compared to women not exposed to childhood trauma. The higher the score in abuse, the higher the emotion dysregulation is.

**Conclusions:**

The main conclusion of this study contributes to the current knowledge on the link between the long-term effects of childhood trauma both to PMDD and to emotion dysregulation. Women with PMDD are more likely to have a history of childhood trauma, which is associated with poorer performance in facial emotion recognition. Trauma, however, is a treatable factor with. Therefore, interventions targeting both to heal trauma and to promote adaptive emotion regulation strategies, could be encouraged to improve the capability of women with a history of childhood trauma to challenge premenstrual symptoms.

**Disclosure of Interest:**

None Declared

